# Multiple Pathways Give a No-Frills Nervous System a Flexible Oxygen Response

**DOI:** 10.1371/journal.pbio.0040306

**Published:** 2006-08-15

**Authors:** Mary Hoff

The tiny nematode *Caenorhabditis elegans* spends most of its life in the soil, searching for an abundance of food and just the right amount of oxygen. But what happens when optimal oxygen and food supplies can’t be found in the same place? More generally, how does the organization of an animal’s neural networks help it produce the right behaviors in competing contexts?

With only 302 neurons, and powerful genetic tools available to the researchers who study it, *C. elegans* is a valuable subject for exploring the neural control of behavior. Previous work has identified just three kinds of neurons as important for sensing and responding to oxygen. These neurons express a family of genes that appear to encode enzymes called soluble guanylate cyclases (sGCs). *C. elegans* sGCs bind oxygen and initiate signaling cascades within the neurons. Animals lacking certain members of this gene family no longer respond normally to oxygen. But, since other neurons also express sGCs, these neurons could play a role in oxygen sensing as well.

*C. elegans*’ response to high ambient oxygen (above 14%) in the presence of food depends on the activity of a neuropeptide receptor called NPR-1. Naturally occurring *npr-1(215F)* nematode strains and laboratory-induced *npr-1(lf)* strains avoid high oxygen whether or not food is present and aggregate in the presence of food. Another naturally occurring strain, *npr-1(215V)*, avoids high oxygen only when food is absent. How does *npr-1(215V)* integrate the information about the two stimuli? To learn the answer, Andy Chang, Cornelia Bargmann, and colleagues systematically assessed the possible role of a number of neurons and genes using mutation and selective gene replacement. Their experiments involved first removing the function of a particular gene (for example, an sGC), then assessing the change in response to oxygen (by looking for changes in the typical distribution of animals along an oxygen gradient), and then finally replacing that gene in only one kind of neuron to see if normal function returns.

Their results revealed some surprises. Previous studies showed that the neurons URX, AQR, and PQR suppress *npr-1(215V)*’s locomotor response to oxygen. In this study, the researchers found another set of neurons—SDQ, ALN, and PLN—expressing sGCs that were able to process information about ambient oxygen levels. They also found that the ion channels OSM-9 and OCR-2 in yet another set of neurons (ADF and ASH) promote high-oxygen avoidance. The researchers concluded that these neurons interact with sGC neurons to produce high-oxygen avoidance and modulation of this response by food.

Another aggregating strain of *C. elegans*, *daf-7*, gave the researchers yet another angle to explore. In crowded, low-food conditions, the developmental gene *daf-7* shows low activity and the nematode enters an alternative larva stage called a dauer. The researchers found that *daf-7* mutants avoided high oxygen with or without food, suggesting that *daf-7*, like *npr-1(215V)*, is involved in suppressing high-oxygen avoidance in the presence of food. Further studies suggested that food might be exerting its influence in part by altering *daf-7* expression in ASI neurons. The researchers also found that *daf-7* mutants expressed higher levels of a gene involved in serotonin synthesis in ADF neurons, suggesting that ADF may represent a convergence point for networks that promote response to high oxygen and those that suppress it.

**Figure:**
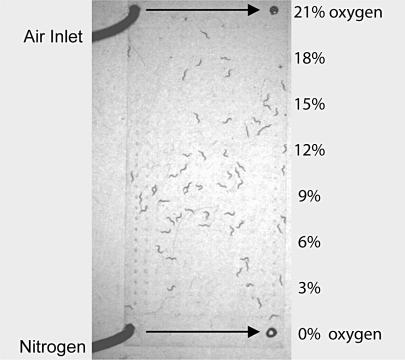
Wild-type *C. elegans* prefers 7%–14% oxygen when placed in an oxygen gradient

The researchers concluded that at least four sets of sensory neurons (some or all of URX, AQR, and PQR; some or all of SDQ, ALN, and PLN; ADF; and ASH) in *C. elegans* promote high-oxygen avoidance, and that these neurons can be suppressed in some cases by other neurons that provide information about food availability. The result is an integrated system that allows this simple organism to respond to its complex environment in an equally complex manner. Electrophysiological examination of other “simple” systems, like motor circuits in the leech and the lobster, has demonstrated comparable complexity in well-defined neural networks, with context-dependent neuronal participation in a particular behavior. The principles uncovered in these systems are likely to be applicable to even more complex brains, whose neuronal circuits are not amenable to comparable dissection.

